# Supplementary material for: Mechanisms governing the pioneering and redistribution capabilities of the non-classical pioneer PU.1
Source: Nat Commun. 2020 Jan 21;11:402. doi: 10.1038/s41467-019-13960-2 (PMC6972792; doi:10.1038/s41467-019-13960-2)
Supplement: Supplementary file 7 — Source data [file 41467_2019_13960_MOESM7_ESM.zip › Source_Data/FigureS2/FigureS2B_MotifScanOutput/KmeansCluster7/homerResults/motif11.similar.html]

motif11

## Information for motif11

G
C
A
T
C
G
T
A
T
C
G
A
G
A
T
C
G
A
T
C
C
G
T
A
A
T
G
C
G
C
T
A
  
Reverse Opposite:  

C
G
A
T
T
A
C
G
G
C
A
T
C
T
A
G
C
T
A
G
A
G
C
T
G
C
A
T
C
G
T
A
  

|  |  |
| --- | --- |
| p-value: | 1e-86 |
| log p-value: | -1.988e+02 |
| Information Content per bp: | 1.627 |
| Number of Target Sequences with motif | 1151.0 |
| Percentage of Target Sequences with motif | 35.86% |
| Number of Background Sequences with motif | 8818.4 |
| Percentage of Background Sequences with motif | 20.17% |
| Average Position of motif in Targets | 99.4 +/- 53.6bp |
| Average Position of motif in Background | 100.7 +/- 70.1bp |
| Strand Bias (log2 ratio + to - strand density) | 0.1 |
| Multiplicity (# of sites on avg that occur together) | 1.20 |
| Motif File: | file (matrix) reverse opposite |

### Similar de novo motifs found

|  |  |  |  |  |  |  |  |
| --- | --- | --- | --- | --- | --- | --- | --- |
| Rank | Match Score | Redundant Motif | P-value | log P-value | % of Targets | % of Background | Motif file |
| 1 | 0.810 | C T G A C A T G T A C G G T C A C G A T A T C G C G A T C A T G C A T G A C G T A C G T C G T A | 1e-80 | -185.650374 | 13.68% | 4.59% | motif file (matrix) |
| 2 | 0.975 | G C A T G C T A C T G A A G T C A G T C C T G A A G T C C G T A C T G A | 1e-78 | -181.676281 | 29.16% | 15.40% | motif file (matrix) |
| 3 | 0.941 | C G T A T C G A A G T C G T A C C T G A A G T C T C G A G T C A C T G A A T C G | 1e-57 | -132.840144 | 17.35% | 8.13% | motif file (matrix) |
| 4 | 0.956 | C G T A C G T A A G T C A G T C C T G A A G T C C T G A | 1e-54 | -124.363130 | 14.58% | 6.44% | motif file (matrix) |
| 5 | 0.896 | A C T G A C G T A C T G A C T G A G C T A C G T C G A T | 1e-49 | -113.157448 | 27.88% | 16.94% | motif file (matrix) |
| 6 | 0.940 | G T A C G C T A G A C T G C T A G T C A G T A C A G T C C G T A G A T C T G C A T C A G | 1e-42 | -97.213583 | 18.29% | 9.95% | motif file (matrix) |
| 7 | 0.756 | A C G T A G T C A C G T A T C G T G C A A T G C T G A C C G T A A G T C C G T A | 1e-15 | -36.779268 | 2.46% | 0.77% | motif file (matrix) |
